# Supplementary material for: A Potential Therapy Using Antisense Oligonucleotides to Treat Autosomal Recessive Polycystic Kidney Disease
Source: J Clin Med. 2023 Feb 10;12(4):1428. doi: 10.3390/jcm12041428 (PMC9966971; doi:10.3390/jcm12041428)
Supplement: Supplementary file 1 [file jcm-12-01428-s001.zip › Figure S1.pdf]

Figure S1

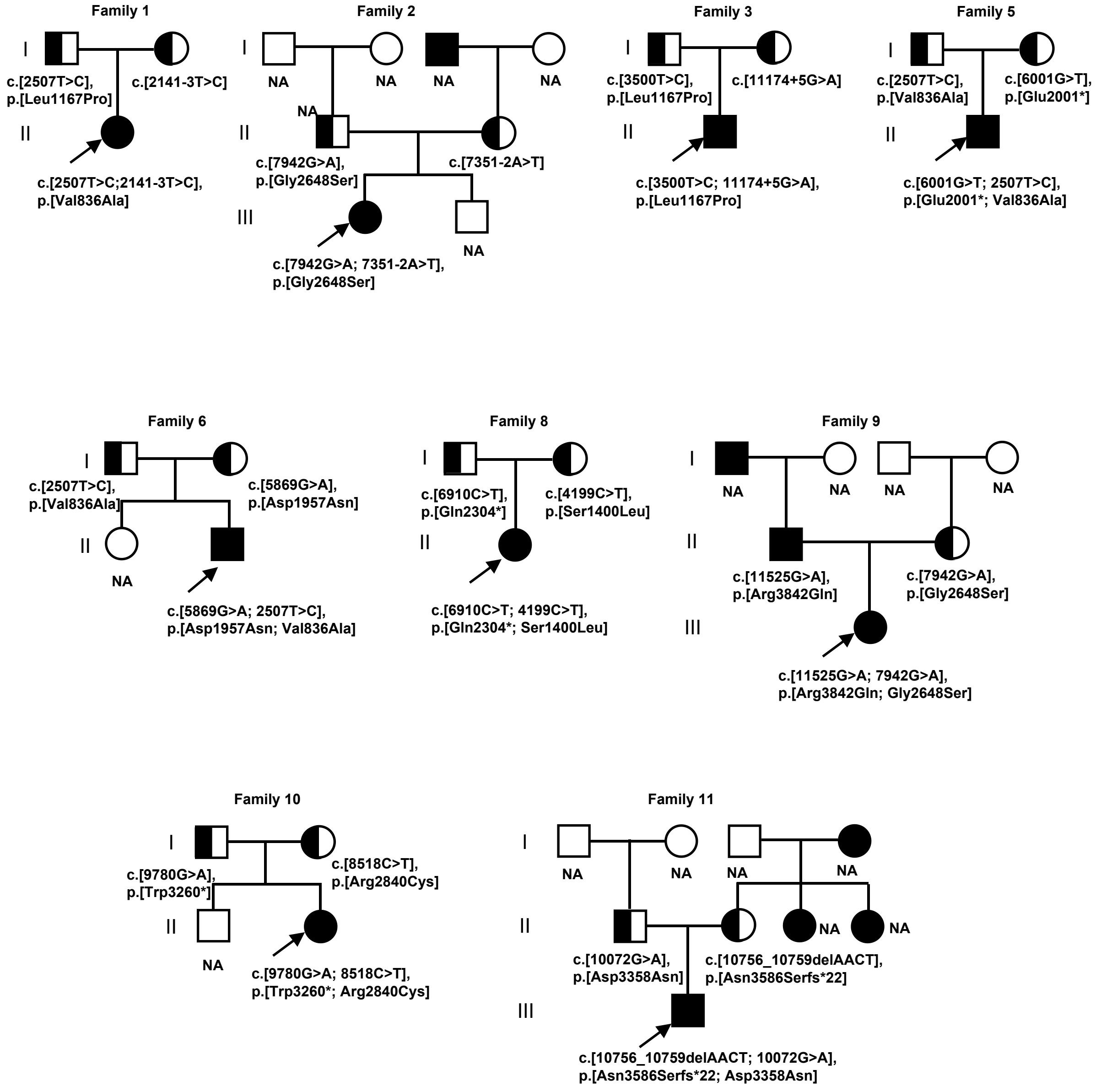

Figure S1. Pedigrees of affected families and variants of the probands. Pedigrees of 9 families with PKHD1 pathogenic variants. Squares indicate males, circles females, filled symbols indicate that the individual presented cysts, open symbols indicate healthy individuals, and half black symbols indicate healthy carrier, NA indicates that DNA sample was unavailable for segregation confirmation. Probands are denoted by arrows.
