# Supplementary material for: A Potential Therapy Using Antisense Oligonucleotides to Treat Autosomal Recessive Polycystic Kidney Disease
Source: J Clin Med. 2023 Feb 10;12(4):1428. doi: 10.3390/jcm12041428 (PMC9966971; doi:10.3390/jcm12041428)
Supplement: Supplementary file 1 [file jcm-12-01428-s001.zip › Figure S2.pdf]

Figure S2

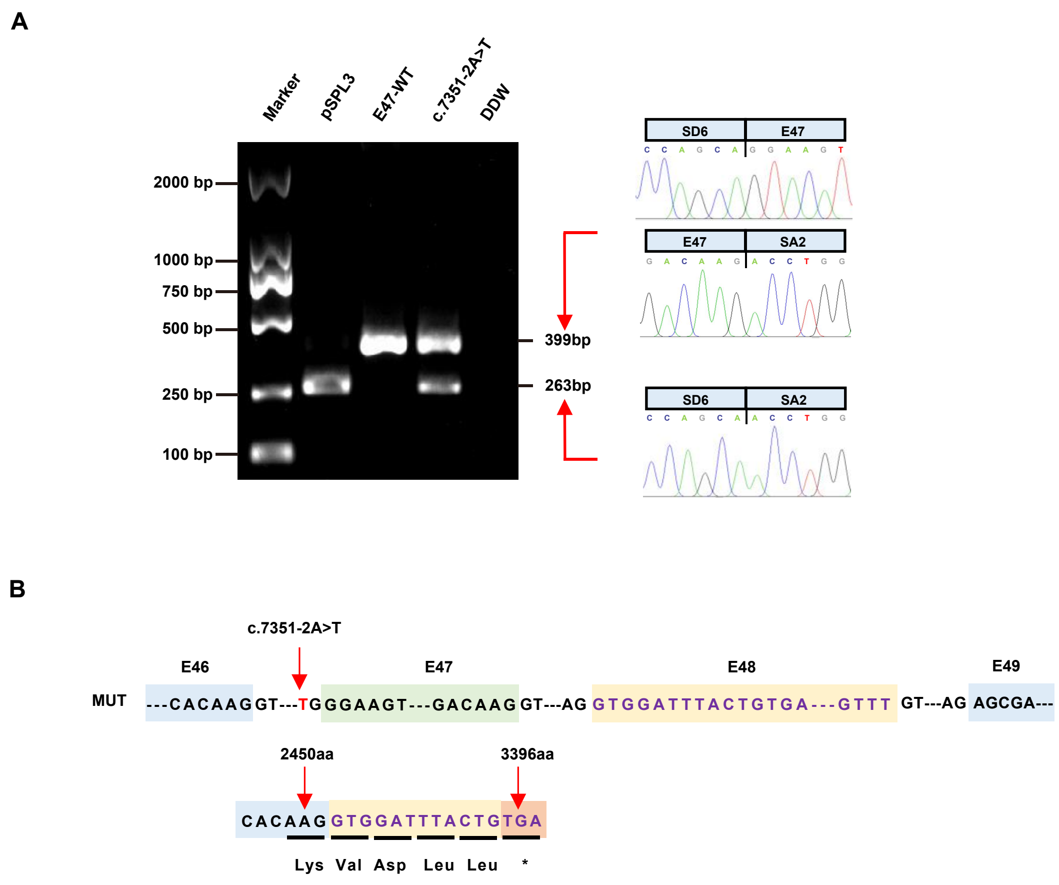

Figure S2. Hybrid minigene assay reveled the aberrant mRNA splicing of classic splicing variant of the *PKHD1* gene in vitro in ARPKD. (A) Agarose gel electrophoresis and directly sequenced (right panel) of the RT–PCR products of minigene transcripts in HEK293T cells of variant c.7351-2A>T. cDNA sequencing of variant c.7351-2A>T showed a exon 47 skipping. Lane 1: marker; Lane 2: pSPL3 (263 bp); Lane 3: E47-WT (399 bp); Lane 4: c.7351-2A>T (399 bp and 263 bp); Lane 5: DDW. (B) Schematic representation of abnormal splicing of the *PKHD1* exons of variant c.7351-2A>T (numbered boxes). A exon 47 skipping in the mutant transcript (upper panel). Predicted amino acids structures translated from the aberrant transcripts of c.7351-2A>T variant deletion of 136bp of exon47 in *PKHD1* (down panel).
