# Supplementary material for: A Potential Therapy Using Antisense Oligonucleotides to Treat Autosomal Recessive Polycystic Kidney Disease
Source: J Clin Med. 2023 Feb 10;12(4):1428. doi: 10.3390/jcm12041428 (PMC9966971; doi:10.3390/jcm12041428)
Supplement: Supplementary file 1 [file jcm-12-01428-s001.zip › Figure S3.pdf]

Figure S3

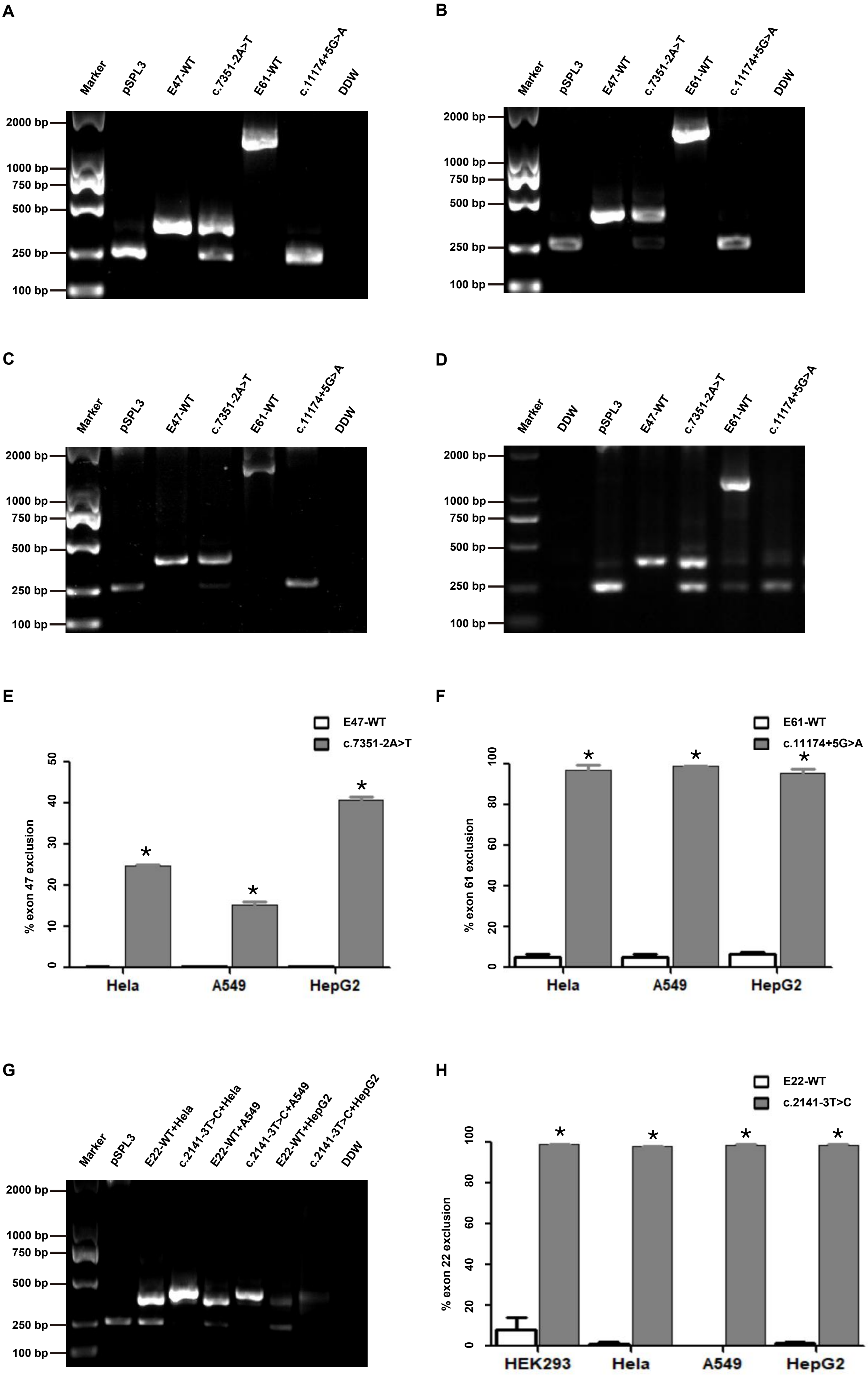
A
Marker
pSPL3
E47-WT
c.7351-2A>T
E61-WT
c.11174+5G>A
DDW
2000 bp
1000 bp
750 bp
500 bp
250 bp
100 bp
B
C
D
E
E47-WT
c.7351-2A>T
% exon 47 exclusion
Hela
A549
HepG2
F
E61-WT
c.11174+5G>A
% exon 61 exclusion
G
E22-WT+Hela
c.2141-3T>C+Hela
E22-WT+A549
c.2141-3T>C+A549
E22-WT+HepG2
c.2141-3T>C+HepG2
H
E22-WT
c.2141-3T>C
% exon 22 exclusion
HEK293

Figure S3. Effect of *PKHD1* gene three splicing variants by Minigene assays in HEK293, HeLa, A549 and HepG2 cells. Hybrid minigene assay reveled the aberrant mRNA splicing in ARPKD of three variants. (A) Gel electrophoresis of the RT-PCR product of minigene transcripts in HEK293 cell of c.7351-2A>T and c.11174+5G>A variant. Lane 1: marker; Lane 2: pSPL3 (263 bp); Lane 3: E47-WT (399 bp); Lane 4: c.7351-2A>T (399 bp and 263 bp); Lane 5: E61-WT (1281 bp); Lane 6: c.11174+5G>A (1281 bp and 263 bp); Lane 7: DDW. (B) Gel electrophoresis of the RT-PCR product of minigene transcripts in HeLa cell of c.7351-2A>T and c.11174+5G>A variant. The lanes, ditto. (C) Gel electrophoresis of the RT-PCR product of minigene transcripts in A549 cell of c.7351-2A>T and c.11174+5G>A variant. The lanes, ditto. (D) Gel electrophoresis of the RT-PCR product of minigene transcripts in and HepG2 cell of c.7351-2A>T and c.11174+5G>A variant. Lane 1: marker; Lane 2: DDW; Lane 3: pSPL3 (263 bp); Lane 4: E47-WT (399 bp); Lane 5: c.7351-2A>T (399 bp and 263 bp); Lane 6: E61-WT (1281 bp); Lane 7: c.11174+5G>A (1281 bp and 263 bp). (E) (F) Quantification of the splicing percentage in the graph was densitometrically calculated on a molar basis as the percentage of exclusion (%) = (lower band/ (lower band+ upper band) *100. Error bars represent SEM (n=3). ***P,0.001, unpaired t-test. The splicing percentage of c.7351-2A>T variant in HeLa, A549 and HepG2 cells (figure S3E). The splicing percentage of c.11174+5G>A variant in HeLa, A549 and HepG2 cells (figure S3F). (G) Gel electrophoresis of the RT-PCR product of minigene transcripts in HEK293 cell of c.2141-3T>C variant. Lane 1: marker; Lane 2: pSPL3 (263 bp); Lane 3: E22-WT-Hela (402 bp and 263bp); Lane 4: c.2141-3T>C-Hela (453 bp); Lane 5: E22-WT-A549 (402 bp and 263bp); Lane 6: c.2141-3T>C-A549 (453 bp); Lane 7: E22-WT -HepG2 (402 bp and 263bp); Lane 8: c.2141-3T>C-HepG2 (453 bp); Lane 9: DDW. (H) The splicing percentage of c.2141-3T>C variant in HEK293, HeLa, A549 and HepG2 cells. Quantification of the splicing percentage in the graph was densitometrically calculated on a molar basis as the percentage of exclusion (%) = (lower band/ (lower band+ upper band) *100. Error bars represent SEM (n=3). ***P,0.001, unpaired t-test.
