# Supplementary material for: A Potential Therapy Using Antisense Oligonucleotides to Treat Autosomal Recessive Polycystic Kidney Disease
Source: J Clin Med. 2023 Feb 10;12(4):1428. doi: 10.3390/jcm12041428 (PMC9966971; doi:10.3390/jcm12041428)
Supplement: Supplementary file 1 [file jcm-12-01428-s001.zip › Figure S4.pdf]

Figure S4

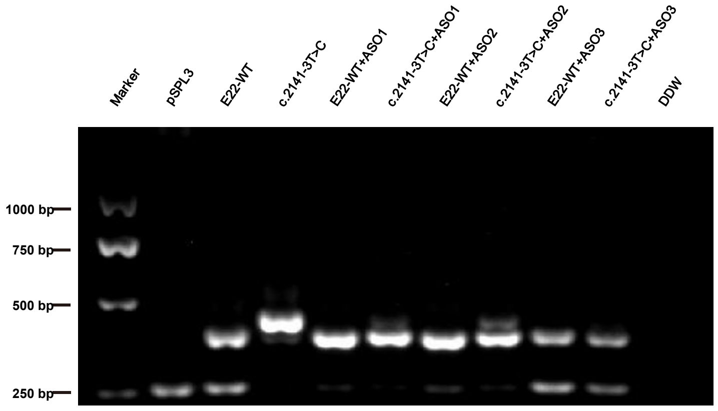

Figure S4. Antisense oligonucleotides (ASOs) rescue the c.2141-3T>C variant of the *PKHD1* gene, which causes aberrant splicing in HEK293T cells. Analysis of splicing correction by RT–PCR upon ASOs treatments. The wild-type (WT) minigene and the corresponding mutant (MU) minigene containing the variant c.2141-3T>C were transfected into HEK293T cells. The three ASOs were then delivered except in the nontreated lanes. The exon elongation caused by c.2141-3T>C was efficiently rescued by ASO1, ASO2 and ASO3.
