# Supplementary material for: A Potential Therapy Using Antisense Oligonucleotides to Treat Autosomal Recessive Polycystic Kidney Disease
Source: J Clin Med. 2023 Feb 10;12(4):1428. doi: 10.3390/jcm12041428 (PMC9966971; doi:10.3390/jcm12041428)
Supplement: Supplementary file 1 [file jcm-12-01428-s001.zip › Table-S1.pdf]

Table S1. Targeted Next-generation sequencing of 180 short related genes.

AHI1	*ALG8*	*ALMS1*	*ANKS3*	*ANKS6*	*ARL13B*	*ARL3*	*ARL6*	*ASS1*	*ATXN10*
B9D1	*B9D2*	*BBIP1*	*BBS1*	*BBS10*	*BBS12*	*BBS2*	*BBS4*	*BBS5*	*BBS7*
BBS9	*C2CD3*	*CC2D2A*	*CCDC28B*	*CCND1*	*CELSR2*	*CEP104*	*CEP120*	*CEP164*	*CEP290*
CEP41	*CEP55*	*CEP83*	*CLUAP1*	*Col4a1*	*CPLANE1*	*CSPP1*	*DCDC2*	*DDX59*	*DNAJB11*
DYNC2H1	*DYNC2I1*	*DYNC2I2*	*DYNC2LI1*	*DYNLT2B*	*DZIP1L*	*FAM111A*	*FAM149B1*	*FAM186B*	*GANAB*
GLI3	*GLIS2*	*HNF1B*	*HSPB11*	*IFT122*	*IFT140*	*IFT172*	*IFT20*	*IFT22*	*IFT27*
IFT43	*IFT52*	*IFT57*	*IFT74*	*IFT80*	*IFT81*	*IFT88*	*INCENP*	*INPP5E*	*INTU*
INVS	*IQCB1*	*KATNIP*	*KIAA0586*	*KIAA0753*	*KIF14*	*KIF7*	*LRP5*	*LZTFL1*	*MAPKBP1*
MKKS	*MKS1*	*MUC1*	*NEK1*	*NEK4*	*NEK8*	*NOTCH2*	*NPHP1*	*NPHP3*	*NPHP4*
OFD1	*PDE6D*	*PIAS1*	*PIBF1*	*PIFO*	*PIK3R4*	*PKD1*	*PKD2*	*PKHD1*	*PMM2*
PRKCSH	*RBM48*	*REN*	*Robo2*	*RPGRIP1L*	*RPS6KA2*	*SCLT1*	*SDCCAG8*	*SEC61A1*	*SEC61B*
SEC63	*SPAG17*	*TBC1D32*	*TCTN1*	*TCTN2*	*TCTN3*	*TMEM107*	*TMEM138*	*TMEM17*	*TMEM216*
TMEM231	*TMEM237*	*TMEM67*	*TRAF3IP1*	*TRIM32*	*TSC1*	*TSC2*	*TTBK2*	*TTC21B*	*TTC26*
TTC30B	*TTC8*	*TXNDC15*	*UMOD*	*VHL*	*WDPCP*	*WDR19*	*WDR35*	*XPNPEP3*	*ZNF423*
