# Supplementary material for: A Potential Therapy Using Antisense Oligonucleotides to Treat Autosomal Recessive Polycystic Kidney Disease
Source: J Clin Med. 2023 Feb 10;12(4):1428. doi: 10.3390/jcm12041428 (PMC9966971; doi:10.3390/jcm12041428)
Supplement: Supplementary file 1 [file jcm-12-01428-s001.zip › Table-S2.pdf]

Table S2. *PKHD1* primers for minigene construct

Exon	Primers(5'-3')
**Exon61- F1**	accagaattctggagctcgagCACTCTCAGTATCTGGCACAATTAGA
**Exon61- R1**	atcaccagatatctgggatccTCTTCCTACATGCTTTTCACCTTAAG
**Exon61- F2**	accagaattctggagctcgagCAATCTTCCTTGCACTCTCAGTATCT
**Exon61- R2**	atcaccagatatctgggatccCATGCTTTTCACCTTAAGGAATCA
**Exon61- F3**	accagaattctggagctcgagCCAGTTATAGATGATGGCAAGAGTG
**Exon61- R3**	atcaccagatatctgggatccCCACCTTGGAAATAGAATAATCATGA
**Exon22- F1**	accagaattctggagctcgagACTGAATGGCTTATCCTCCTGC
**Exon22- R1**	atcaccagatatctgggatccGTGAATCTGGTGCTGCATTCTCA
**Exon22- F2**	accagaattctggagctcgagGGCAGCAGATAAGTAGGAATCTATGC
**Exon22- R2**	atcaccagatatctgggatccGAAACTCTGACAGTAGGCCCAGTAA
**Exon22- F3**	accagaattctggagctcgagACTAAGCTCTCCTTGTCAGATTGATTG
**Exon22- R3**	atcaccagatatctgggatccCTGACTCGGAATTAGTTCTCAAAGC
**Exon47- F1**	accagaattctggagctcgagGGTCCTTTGTCATATGTGTGATTGG
**Exon47- R1**	atcaccagatatctgggatccGAATGTGATAGGCACCATAATAGGAG
**Exon47- F2**	accagaattctggagctcgagTCTCTTAGTCAATAGCTCGCTTTTTT
**Exon47- R2**	atcaccagatatctgggatccGCAACAGTTTCTACTGATGCAATCT
