# Supplementary material for: A Potential Therapy Using Antisense Oligonucleotides to Treat Autosomal Recessive Polycystic Kidney Disease
Source: J Clin Med. 2023 Feb 10;12(4):1428. doi: 10.3390/jcm12041428 (PMC9966971; doi:10.3390/jcm12041428)
Supplement: Supplementary file 1 [file jcm-12-01428-s001.zip › Table-S3.pdf]

Table S3. Prediction of nine missense variants of *PKHD1*.

Exon	Nucleotide Change	Amino Acid Change	PolyPhen-2	PROVEAN	SIFT	Mutation Taster	ACMG
5	c.325G>A	Ala109Thr	P (0.156)	Neutral (-0.075)	Tolerated (0.13)	D (0.99)	LP (PS2+PM2)
24	c.2507T>C	Val836Ala	D (0.991)	Deleterious (-2.973)	Affect protein function (0.000)	N (0.67)	VUS (PM2+PP3+PM3)
30	c.3500T>C	Leu1167Pro	B (0.214)	Deleterious (-4.590)	Affect protein function (0.000)	N (0.99)	VUS (PM2+PP3)
32	c.4199C>T	Ser1400Leu	P (0.634)	Deleterious (-4.938)	Affect protein function (0.000)	D (0.90)	VUS (PM2+PP3)
36	c.5869G>A	Asp1957Asn	P (0.733)	Deleterious (-3.664)	Affect protein function (0.040)	D (0.96)	LP (PS2+PM2+PP3)
38	c.6245C>T	Thr2082Ile	B (0.001)	Deleterious (-3.091)	Affect protein function (0.02)	D (0.99)	VUS (PM2+PP3)
50	c.7942G>A	Gly2648Ser	D (1.000)	Neutral (-2.147)	Tolerated (0.35)	D (0.84)	VUS (PM2)
54	c.8518C>T	Arg2840Cys	D (0.999)	Deleterious (-3.263)	Tolerated (0.06)	D (0.61)	LP (PS1+PM2+PM3+PP3)
60	c.10072G>A	Asp3358Asn	D (1.000)	Deleterious (-4.148)	Affect protein function (0.000)	D (0.97)	LP (PS2+PM2+PM3+PP3)
65	c.11525G>A	Arg3842Gln	D (1.000)	Neutral (-0.972)	Affect protein function (0.000)	N (0.95)	VUS (PM2+PP3)

PolyPhen-2 prediction score, D: Probably damaging (>=0.909), P: Possibly damaging (0.447<=P<=0.909), B: Benign (<=0.446))

PROVEAN, -14~-2.5:Deleterious, -2.5~14:Neutral

SIFT, sorting intolerant from tolerant; sift≥0.05,T, tolerated; sift < 0.05, D, deleterious

Mutation Taster, A: disease_causing_automatic, D: disease_causing, N: polymorphism, P:polymorphism_automatic
