# Supplementary material for: A Potential Therapy Using Antisense Oligonucleotides to Treat Autosomal Recessive Polycystic Kidney Disease
Source: J Clin Med. 2023 Feb 10;12(4):1428. doi: 10.3390/jcm12041428 (PMC9966971; doi:10.3390/jcm12041428)
Supplement: Supplementary file 1 [file jcm-12-01428-s001.zip › Table-S4.pdf]

Table S4. Prediction of three splice site variants of *PKHD1*.

Exon	Nucleotide Change	The Human Splicing Finder	SpliceAI	MaxEntScan
22	c.2141-3T>C	NO significant impact on splicing signals	Get new WT Acceptor site	cut-off < 3.0
47	c.7351-2A>T	broken WT Acceptor site<br>decreasing the score from 86.45 to 58.58	broken WT Acceptor site	cut-off ≥ 3.0
61	c.11174+5G>A	broken WT Donor site<br>decreasing the score from 92.39 to 82.83	broken WT Donor site	cut-off ≥ 3.0
